# Uniform Surface Modification of 3D Bioglass^®^-Based Scaffolds with Mesoporous Silica Particles (MCM-41) for Enhancing Drug Delivery Capability

**DOI:** 10.3389/fbioe.2015.00177

**Published:** 2015-11-06

**Authors:** Elena Boccardi, Anahí Philippart, Judith A. Juhasz-Bortuzzo, Ana M. Beltrán, Giorgia Novajra, Chiara Vitale-Brovarone, Erdmann Spiecker, Aldo R. Boccaccini

**Affiliations:** ^1^Institute of Biomaterials, Department of Materials Science and Engineering, Friedrich-Alexander University Erlangen-Nürnberg, Erlangen, Germany; ^2^Center for Nanoanalysis and Electron Microscopy (CENEM), Institute of Micro- and Nanostructure Research, Department of Materials Science and Engineering, Friedrich-Alexander University Erlangen-Nürnberg, Erlangen, Germany; ^3^Institute of Materials Physics and Engineering, Applied Science and Technology Department, Politecnico di Torino, Turin, Italy

**Keywords:** ordered mesoporosity, silica, MCM-41, bioactive glass, scaffolds, drug release, ibuprofen

## Abstract

The design and characterization of a new family of multifunctional scaffolds based on bioactive glass (BG) of 45S5 composition for bone tissue engineering and drug delivery applications are presented. These BG-based scaffolds are developed via a replication method of polyurethane packaging foam. In order to increase the therapeutic functionality, the scaffolds were coated with mesoporous silica particles (MCM-41), which act as an *in situ* drug delivery system. These sub-micron spheres are characterized by large surface area and pore volume with a narrow pore diameter distribution. The solution used for the synthesis of the silica mesoporous particles was designed to obtain a high-ordered mesoporous structure and spherical shape – both are key factors for achieving the desired controlled drug release. The MCM-41 particles were synthesized directly inside the BG-based scaffolds, and the drug-release capability of this combined system was evaluated. Moreover, the effect of MCM-41 particle coating on the bioactivity of the BG-based scaffolds was assessed. The results indicate that it is possible to obtain a multifunctional scaffold system characterized by high and interconnected porosity, high bioactivity, and sustained drug delivery capability.

## Introduction

**Figure 1:**
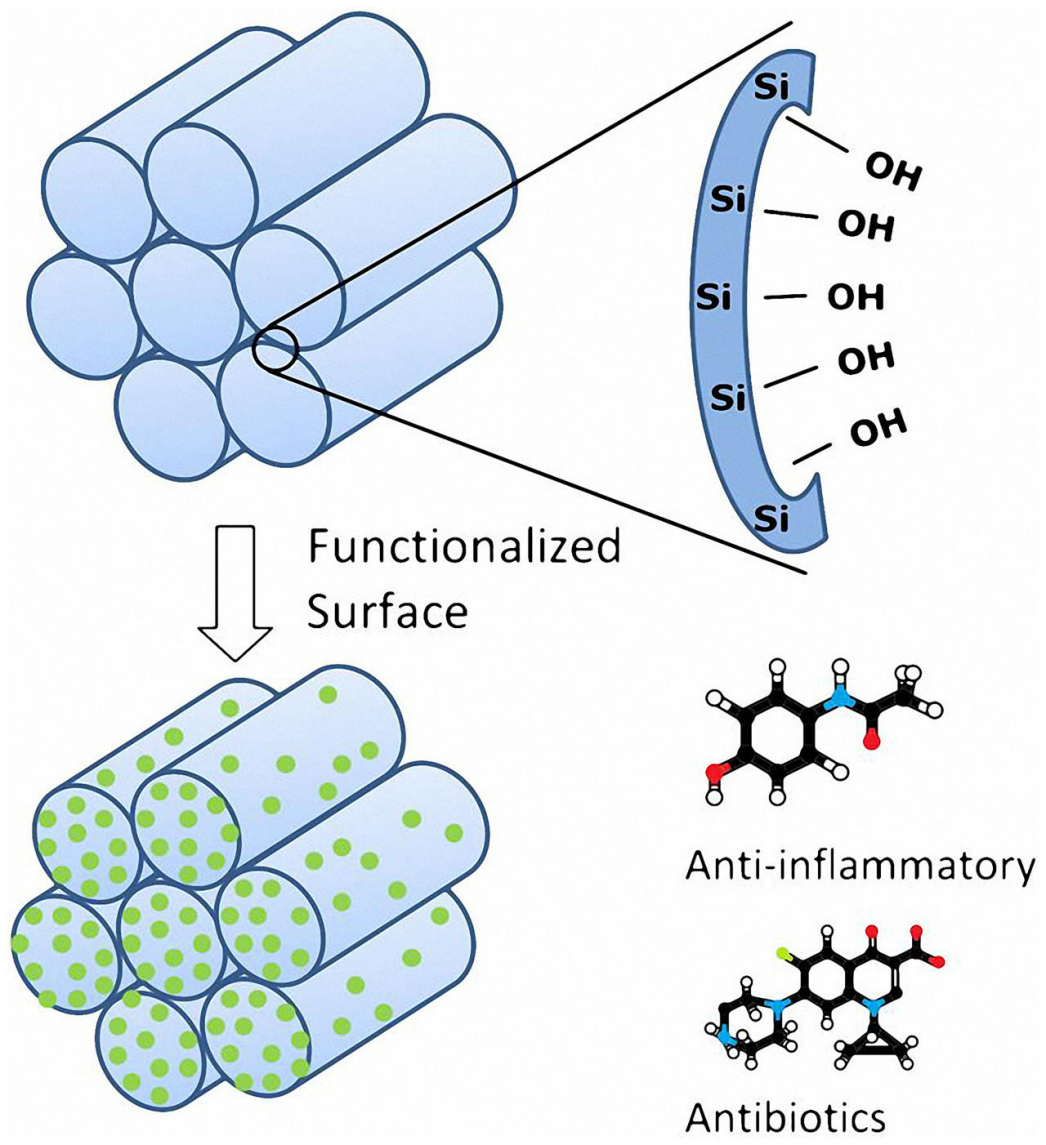
**Main features of the mesoporous silica materials (figure modified from Vallet-Regí, 2006b)**.

One of the most promising fields of tissue engineering is the development of porous 3D engineered scaffolds to enhance bone regeneration and neovascularization (Porter et al., 2009). The main challenge is the design of materials able to match at the same time the biological and the mechanical properties of the natural bone tissue (Mastrogiacomo et al., 2006; Stevens, 2008; Philippart et al., 2015). However, the design of the scaffolds is not the only challenge, in fact the first problem after implantation is the exposure to inflammatory and infection risks with further complications, e.g., septicemia and potential implant failure (Misch and Wang, 2008). To avoid these consequences, a large amount of antibiotics and anti-inflammatory drugs are administered to the patient, which can increase the healing time, the stay at the hospital, and costs (Neut et al., 2003). Nowadays, the most popular ways for drug intake are oral administration and injection. However, these methods may be affected by a lack of efficiency especially since the release of the drug is not targeted to the area that needs to be treated (Vallet-Regí et al., 2012a). For all these reasons, the development of local drug-release systems, which enable controlled release kinetics, has increased considerably during the past few years (Vallet-Regí, 2006a; Slowing et al., 2007; Cotí et al., 2009; Vitale-Brovarone et al., 2009; Wu et al., 2013). In this context, the combination of bioactive scaffolds with local drug delivery carriers is gaining increasing research efforts in the bone tissue engineering field (Philippart et al., 2015). Several matrices have been tested so far, such as organic polymers, organic–inorganic hybrid materials, bioactive glasses (BG), and ceramics (Wu and Chang, 2014). One approach gaining increasing interest involves obtaining drug carriers that are structured at the nanoscale. Since 1992, when silica-based MCM-41 was developed (Mobil Composition of Matter No. 41) (Beck et al., 1992), highly ordered mesoporous materials have attracted the attention of many scientists and in 2001 they were proposed as drug delivery system (Vallet-Regí et al., 2001). The most interesting features of these materials are the regular pore system, high specific surface area and high pore volume (Vallet-Regí et al., 2001, 2012a,b; Vallet-Regí, 2006b; Zhao et al., 2013). These silica-based mesoporous materials are able to incorporate relatively high content of drugs into the mesopores. Moreover, their silanol groups can be functionalized (Figure 1) and the pore diameter can be modulated, allowing a better control of the drug-release kinetic (Grün et al., 1997; Vallet-Regí et al., 2001, 2012b; Vallet-Regí, 2006b; Wu and Chang, 2014). Two mechanisms have been proposed to describe mesoporous silica material formation. The first model describes the addition of silicate to micelles formed using *n*-decyltrimethylammonium bromide. In this way, the silica precursor polymerizes around the already formed micelles (Zhao et al., 2013). The second proposed mechanism is that the addition of the silica precursor to an aqueous *n*-decyltrimethylammonium bromide solution induces the ordering of silica-encased surfactant micelles simultaneously. In this case, the micelle formation requires the silica precursor to be present (Vallet-Regí et al., 2012a; Zhao et al., 2013).

MCM-41 has become the most popular member of the mesoporous silicate materials family, and it has been considered also as drug carrier.(Vallet-Regí et al., 2012b) Nowadays, it is possible to find in literature different approaches for the synthesis of spherical MCM-41 (Grün et al., 1999; Cai et al., 2001; Zeleňák et al., 2008; Liu et al., 2009). Grün et al. (1997, 1999) proposed a novel pathway for the production of spherical MCM-41 applying a modification of the Stöber reaction (Stober and Fink, 1968) for the synthesis of spherical non-porous silica particles. The approach involves introducing a low-boiling alcohol, such as ethanol or isopropanol, as co-solvent for the silica source in order to get a more homogeneous solution (Grün et al., 1999). Starting from the work of Grün et al. (1999), it is possible to obtain well-shaped spherical particles; however, the mesoporosity is not homogeneously present. On the other hand, following a standard procedure reported by Zeleňák et al. (2008), it is possible to obtain well-ordered mesoporous structures; however, the particles are not spherical and the size distribution is usually broad. Combining these two synthesis pathways, a new solution for the synthesis of spherical mesoporous silica particles has been proposed in this study. Thus, the aim of the present work is the synthesis of spherical silica mesoporous particles (MCM-41) inside porous BG-based scaffolds [45S5 BG composition (Hench, 2015)] to combine in the same system the drug uptake and release capabilities of this mesoporous material with the bioactivity properties of the BG. The concept of incorporating a silica drug carrier into bioactive silicate scaffolds has been previously explored (Mortera et al., 2008); however, the main advantage of the approach introduced in this paper is the possibility to obtain a highly homogeneous coating of the BG scaffold struts with highly ordered mesoporous silica particles without affecting the BG bioactivity. Moreover, the total amount of produced particles obtained per single batch increases by ~60% combining the two standard procedures reported in literature (Grün et al., 1999; Zeleňák et al., 2008), which represents another advantage of the present approach.

## Materials and Methods

### MCM-41 Particle Synthesis

The procedure adopted to prepare MCM-41 was a combination of the standard pathway for the production of mesoporous silica particles and a modification of the Stöber reaction (Stober and Fink, 1968) for the preparation of non-porous silica spheres proposed by Grün et al. (1999). In this way, the reaction took place in a more homogeneous environment, resulting in the formation of sub-micron sized spherical MCM-41 particles and the total amount of the cationic surfactant, which is extremely toxic, can be reduced (Grün et al., 1999). A low-boiling alcohol such as ethanol was added as co-solvent for the tetra-*n*-alkoxysilane to make it soluble. The reactants were ammonia (catalyst of the reaction), *n*-hexadecyltrimethylammonium bromide (CTAB, surfactant), pure ethanol (co-solvent of silica source), and tetraethyl orthosilicate (TEOS), all purchased from Sigma-Aldrich (Germany). Pure ethanol and ammonia (28–30 wt.%) solution were mixed with deionized water. The cationic surfactant was added to the solution under continuous stirring for 20 min. Once the solution was clear, TEOS was added (0.25 mL min^−1^). All synthesis steps were carried out at room temperature (RT), which is the optimal temperature condition for the reaction with cationic surfactant in basic conditions as reported by Zhao et al. (2013). After 2 h of stirring, the resulting dispersion was centrifuged and washed once with deionized water and twice with ethanol in order to remove completely every trace of ammonia, collected in a ceramic crucible, dried, and calcined in air. The solutions used are reported in Table 1. For samples MCM-41_A (Zeleňák et al., 2008), MCM-41_B, and MCM-41_C, the thermal treatment was 60°C (2°C min^−1^) for 12 h and 550°C (2°C min^−1^) for 6 h; for sample MCM-41_D (Grün et al., 1999), the thermal treatment was 90°C (2°C min^−1^) for 12 h and 550°C (1°C min^−1^) for 5 h.

**Table 1:** **Composition of four different synthesis solutions used for the preparation of mesoporous silica particles**.

Sample	H_2_O (mL)	EtOH (mL)	NH_3_ (mL)	CTAB (g)	TEOS (mL)
MCM-41_A (Zeleňák et al., 2008)	29	–	18.5	0.2	1
MCM-41_B	11	18	18.5	0.2	1
MCM-41_C	4	25	18.5	0.2	1
MCM-41_D (Grün et al., 1999)	11	19	3.3	0.62	1.25

### Scaffolds Preparation

The template used to prepare 3D porous scaffolds was polyurethane (PU) packaging foams (45 ppi) (Eurofoam Deutschland GmbH Schaumstoffe). BG powder (particle size 5 μm of 45S5 composition) was used. 45S5 BG-based scaffolds were produced by the replica technique, according to the method described by Chen et al. (2006). Briefly, the slurry for the scaffolds fabrication was prepared by dissolving polyvinyl alcohol (PVA) in deionized water at 80°C for 1 h, the concentration being 0.01 mol L^−1^. Then, 45S5 BG powder was added to 25 mL PVA–water solution to obtain a concentration of 40 wt.%. Each procedure was carried out under vigorous stirring using a magnetic stirrer for 1 h. The sacrificial PU templates, cut to cylinders (7 mm in height and 5 mm in diameter), were immersed in the slurry for 10 min. The foams were retrieved and the extra slurry was completely squeezed out manually. The samples were then dried at RT for at least 12 h. The dip coating in the slurry was repeated three times to increase the coating thickness and consequently the mechanical properties. After the second and third coating, the extra slurry was completely removed using compressed air as explained elsewhere (Boccardi et al., 2015). Post-foaming heat treatment for the burning-out of the sacrificial template and sintering of the BG structure was programed. The burning and sintering conditions were: 400°C for 1 h and 1050°C for 1 h, respectively. The heating and cooling rates were 2 and 5°C min^−1^, respectively.

### Composite System Preparation

**Figure 2:**
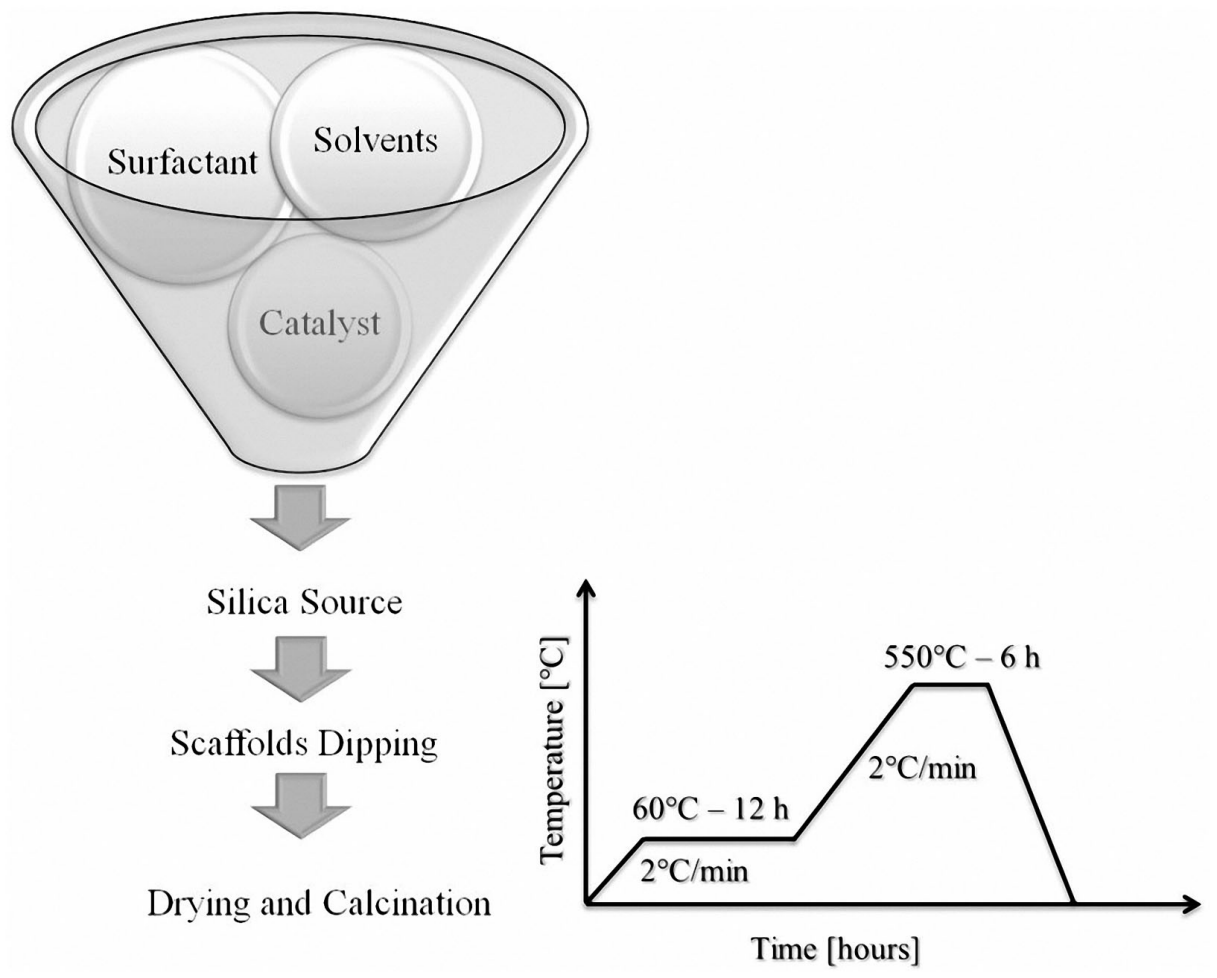
**Scheme of the coating of BG-based scaffolds with mesoporous silica particles and heat-treatment schedule used**.

MCM-41_A, MCM-41_B, and MCM-41_D samples were used for the preparation of BG_MCM-41 composite scaffolds. MCM-41_C solution was not used because it did not show any ordered mesoporosity. The coating procedure used here was similar to the one reported by Mortera et al. (2008). The procedure consisted of four steps, i.e., hydrolysis of TEOS in MCM-41-synthesis solution, dipping of scaffolds for particles impregnation, drying of the scaffolds, and calcination (heat treatment) for the removal of the surfactant. After TEOS addition, the solution was stirred for 10 min to promote the hydrolysis of the silica precursor. Scaffolds were then immersed in the silica synthesis batch for 10 min and meanwhile the solution was kept under vigorous stirring in order to enhance the coating of the inner core of the BG scaffolds. The resulting BG-based scaffolds coated with the MCM-41 particles were heat-treated at 60°C (2°C min^−1^) for 12 h for drying and at 550°C (2°C min^−1^) for 6 h in air as reported in Figure 2.

### Drug-Release Test

To load silica particles with a drug, Ibuprofen (>98%, purchased from Sigma-Aldrich) as model drug was dissolved in hexane (33 mg mL^−1^) and MCM-41 particles were added to the drug solution (33 mg mL^−1^) at RT following the procedure presented in literature (Vallet-Regí et al., 2001). The samples with the drug solution were then placed in a vacuum hood at RT at 300 mbar for 10 min in order to enhance the drug infiltration inside the mesoporosity. After 12 h, this procedure was repeated, the drug solution was removed and the particles were dried in a vacuum hood at RT. All the particle synthesis solutions were tested for their drug-releasing capability.

The scaffolds coated with MCM-41_B were in contact with the drug solution for 3 days (10 mg mL^−1^) before the starting of the drug-release test, in order to get a better infiltration of the drug. Only the scaffolds coated with MCM-41_B particles were considered for the test, because the resulting particles were still spherical and with ordered mesoporosity and the results were compared with those obtained on scaffolds not coated with MCM-41.

The drug-release kinetics from all samples, particles (10 mg each sample), and scaffolds was assessed by soaking the samples in 4 mL of PBS, kept at 37°C until the complete release of ibuprofen. At every time point, 1 mL of solution was uptake for the drug-release analysis and substituted with 1 mL of fresh PBS. A UV-vis spectrophotometer was used to evaluate the amount of released drug. The calibration curve was calculated using a solution of ibuprofen in PBS with different known concentrations, on the basis of the absorption at 273 nm, typical of this molecule (Vallet-Regí et al., 2001).

### Bioactivity and Stability of the Composite System

Simulated body fluid (SBF) was prepared by dissolving reagent-grade 8.035 g L^−1^ NaCl, 0.355 g L^−1^ NaHCO_3_, 0.225 g L^1^ KCl, 0.231 g L^−1^ K_2_HPO_4_ (3H_2_O), 0.311 g L^−1^ MgCl_2_ (6H_2_O), 0.292 g L^−1^ CaCl_2_, and 0.072 g L^−1^ Na_2_SO_4_ in deionized water and buffered at pH 7.4 at 36.5°C with 6.118 g L^−1^ tris(hydroxymethyl) aminomethane [(CH_2_OH)_3_CNH_2_] and 1M HCl, as previously reported by Kokubo and Takadama (2007). Cylindrical BG foams coated and not coated with mesoporous silica particles were immersed in SBF at a 1.5 g L^−1^ ratio (Cerruti et al., 2005). The stability of the MCM-41 coating was evaluated in Tris-buffered solution [tris(hydroxymethyl) aminomethane]. Only the scaffolds coated with MCM-41_B were tested, because the silica particles showed suitable features in terms of homogeneous coating, shape, and ordered mesoporosity. In both cases, the solution was kept in a polystyrene container at 37°C in a shaking incubator (90 rpm) up to 1 week. The solution was renewed every 2 days in order to better mimic the *in vivo* behavior, as carried out also in previous studies (Chen et al., 2006). At the end of the incubator period, the foams were washed with deionized water, dried, and stored for further characterizations.

### Characterization Techniques

The shape and the surface structure of the resulting MCM-41 particles and BG_MCM-41 were evaluated by means of scanning electron microscope (SEM) (Auriga 0750 from ZEISS). The porous structure of the particles was assessed with high-resolution transmission electron microscopy (HRTEM) (Phillips CM30) operating at an acceleration voltage of 300 kV. For the TEM observation, the samples were dispersed with ethanol on a lacey carbon film. The pore diameter analyses were conducted on HRTEM images with ImageJ analysis software. Small angle X-ray diffraction (SAXRD), carried out using Philips Xpert Diffractometer, was used to analyze the porous structure and the pore diameter of the silica particles. Diffraction data were recorded between 1 and 10° 2θ at an interval of 0.02° 2θ. Nitrogen adsorption desorption analysis was conducted at 77 K in a Quantachrome Autosorb Instrument to assess the specific surface area and the pore size of the particles. Prior to the measurements, the samples were outgassed for 12 h at 300°C under vacuum. The specific surface area and pore size of MCM-41 microspheres were evaluated, respectively, with BET method and BJH method.

## Results

### MCM-41 Particles

**Figure 3:**
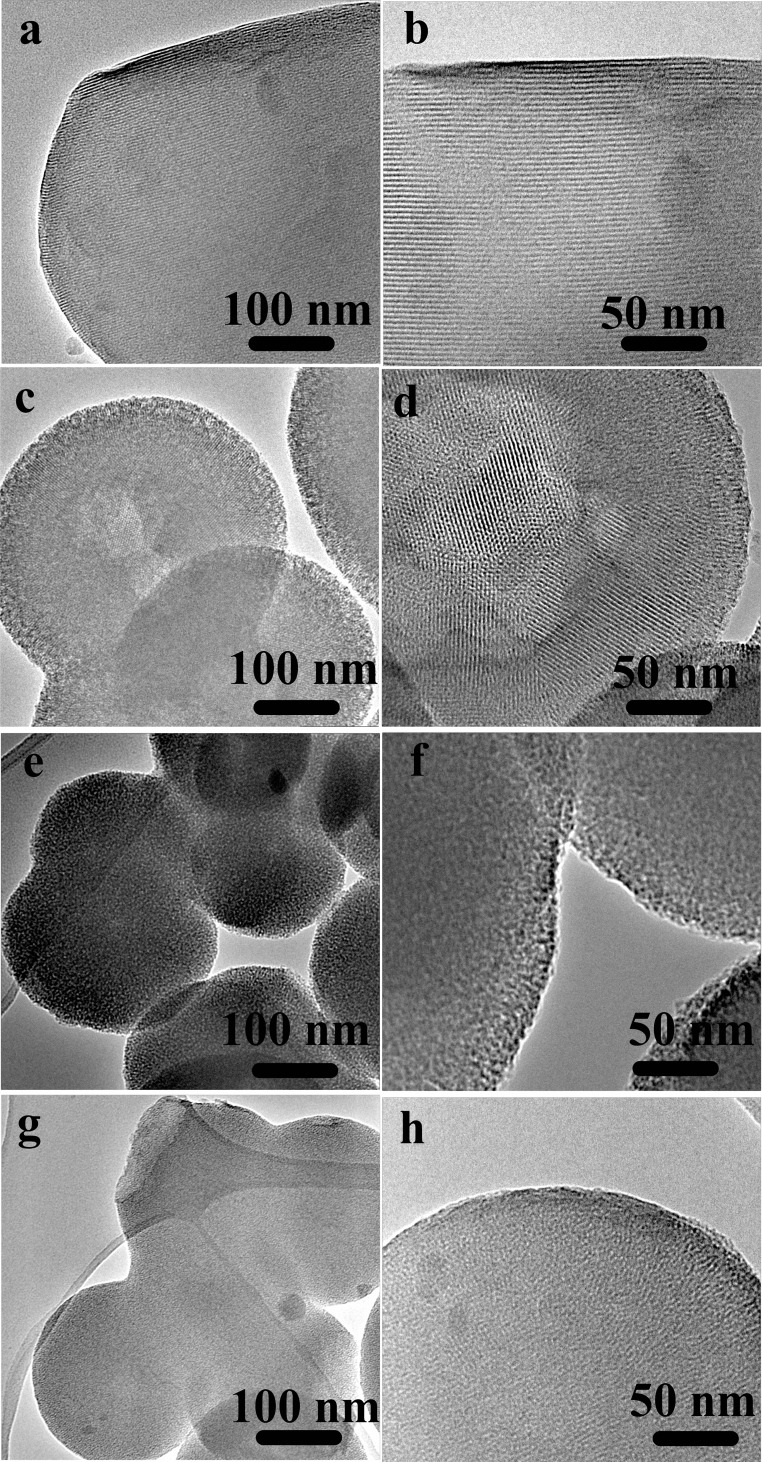
**HRTEM images of sample MCM-41_A (a,b) and sample MCM-41_B (c,d), which are characterized by ordered mesoporosity, sample MCM-41_C (e,f) and MCM-41_D (g,h), which are characterized by a disordered porosity**.

**Figure 4:**
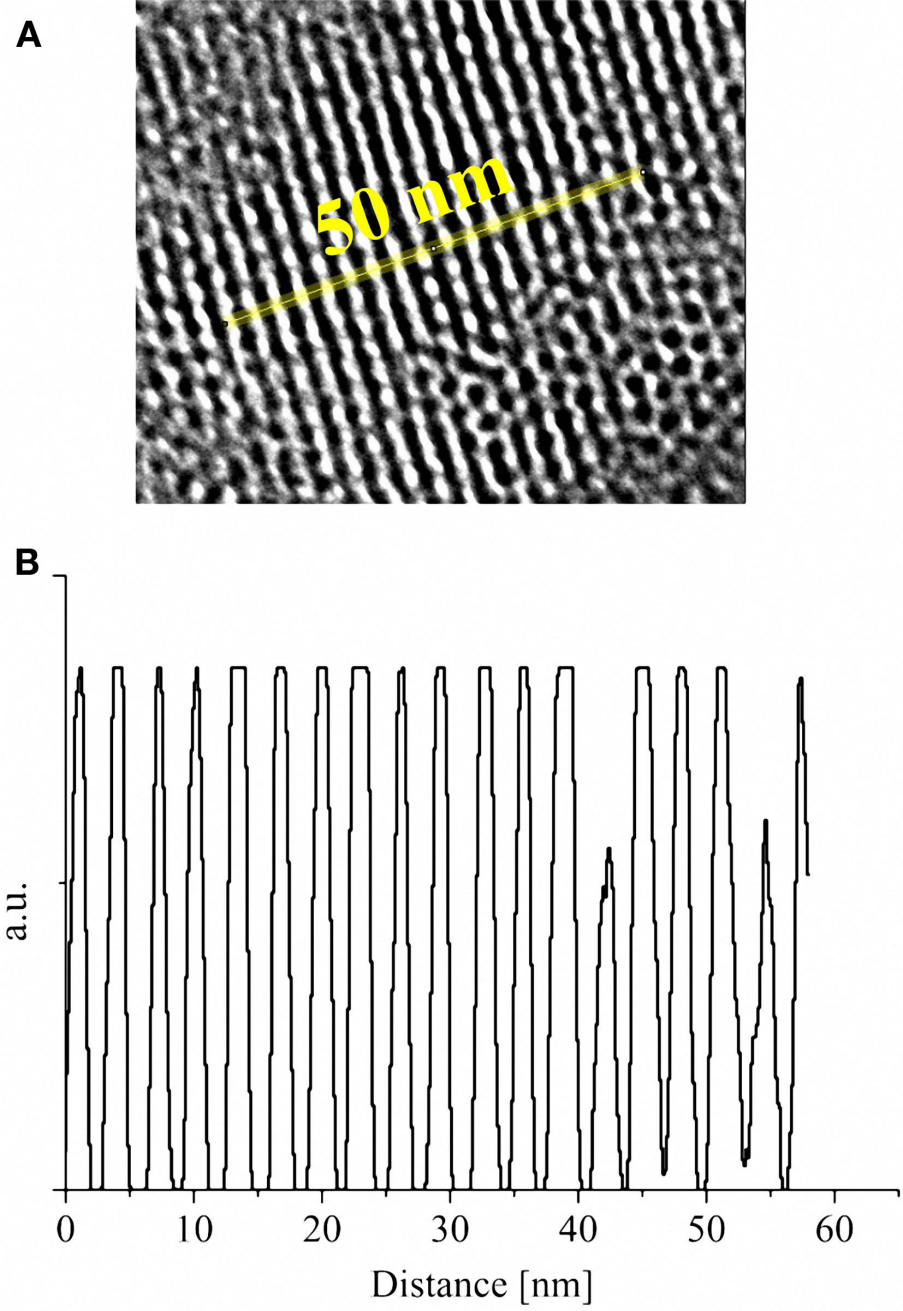
**High-resolution image of the ordered mesoporous structure of MCM-41_B after analysis with FFT and inverse FFT (A); plot of the distance between the pore channels obtained with ImageJ plug in plot applied along the yellow line (B)**.

The morphology and the microstructure of the obtained MCM-41 particles were assessed by HRTEM micrographs. HRTEM images of sample MCM-41_A and MCM-41_B showed the existence of highly ordered hexagonal array and streaks structural features (Figures 3a–d). The hexagonal array and the streaks are the view of the crystals whose axes are, respectively, parallel and perpendicular to the line of vision. Sample MCM-41_C, which was prepared with a high concentration of ethanol in the synthesis solution, was porous however the porosity was not ordered (Figures 3e,f). Moreover MCM-41_D particles were porous but the porosity was not completely ordered, in contrast with the results reported in literature (Grün et al., 1999) (Figures 3g,h). From the analysis of the HRTEM images with ImageJ analysis software, the dimension of the pores was evaluated, which was found to be around 3 nm for all samples (Figure 4). The analysis has been done applying the Fast Fourier Transform (FFT) and the inverse FFT (Figure 4A) to the image, and the plug in plot (Figure 4B) has been used to evaluate the distance between the pore channels.

**Figure 5:**
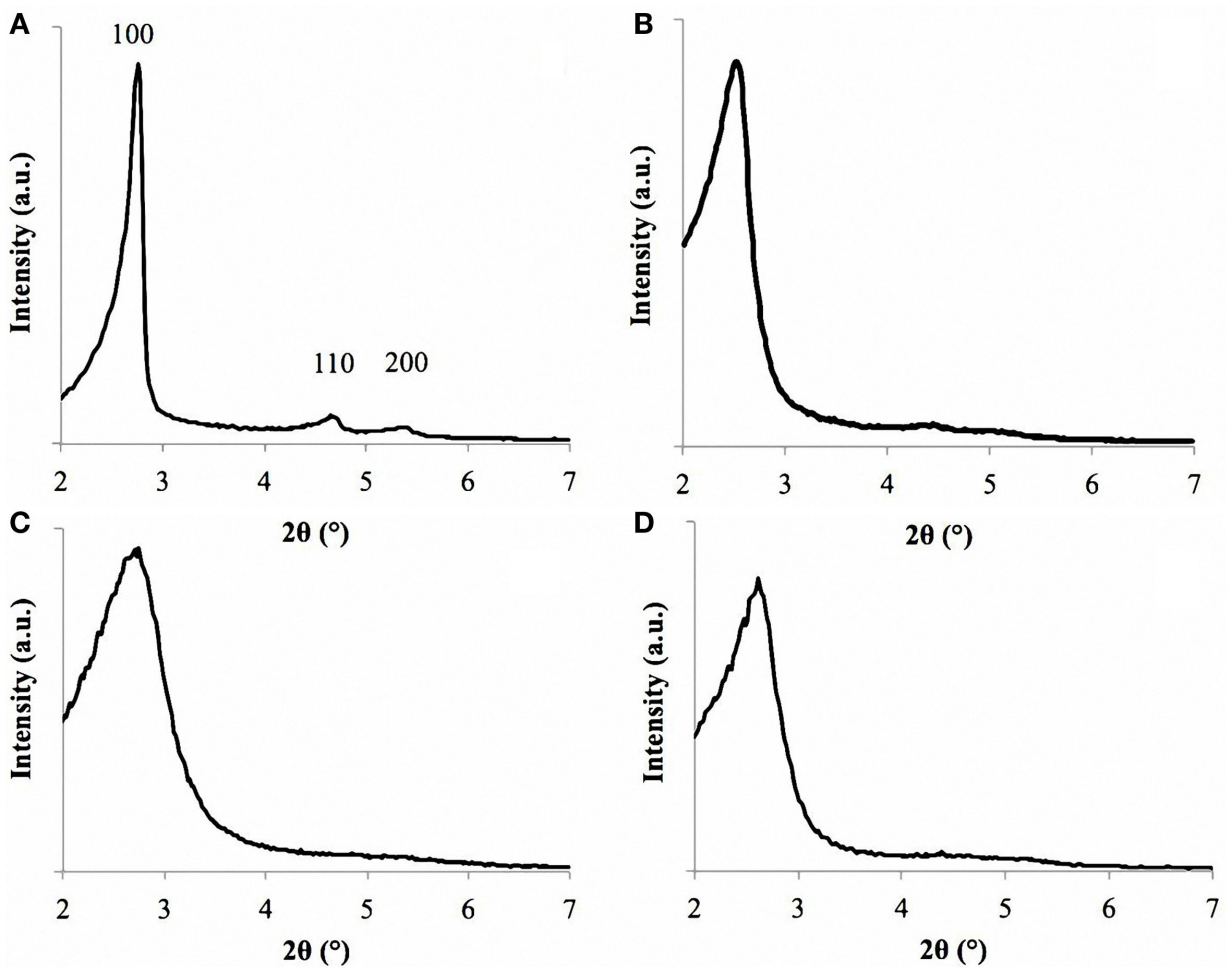
**SAXRD of sample MCM-41_A (A), MCM-41_B (B), MCM-41_C (C), and MCM-41_D (D)**. Sample MCM-41_A is characterized by the three peaks, labeled as 100, 110, and 200.

The pore size dimensions were confirmed also by SAXRD analysis. The spectra of sample MCM-41_A exhibited three sharp peaks, called Bragg peaks, indicating the long-range order present in the material, which is typical of MCM-41 materials (Vallet-Regí et al., 2001) (Figure 5A) in agreement with literature (Grün et al., 1999). These peaks arise from the quasi-regular arrangement of the mesopores in the bulk material (Grün et al., 1999; Vallet-Regí et al., 2001). The Bragg peaks can be indexed assuming a hexagonal symmetry. *2 theta* values of sample MCM-41_A namely 2.75, 4.65, and 5.10 can be indexed as (100), (110), and (200) reflections, respectively. These values were close to those reported by Grün et al. (1999). The repeating distance, *a*_0_, between two pore centers may be calculated by *a*_0_ = (2/√3)*d*_100_. The pore diameter can be evaluated from *a*_0_ subtracting 1.0 nm, which is approximately the value of the pore wall thickness (Grün et al., 1999). For MCM-41_B particles, it was possible to identify unequivocally only the main peak (100) (Figure 5B), meanwhile the 110 and 200 peaks were less pronounced but still visible. The SAXRD results combined with HRTEM results confirm thus the mesoporous ordered structures of MCM-41_A and MCM-41_B particles.

For samples MCM-41_C and MCM-41_D, only the main peak (100) was identified, in agreement with the HRTEM analysis (Figures 5C,D). The first peak is in fact an indicator of the presence of mesoporosity in the sample. Also in this case, it was possible to evaluate the pore diameter with the Bragg’s law and the resulting values were in agreement with the ImageJ analysis.

**Figure 6:**
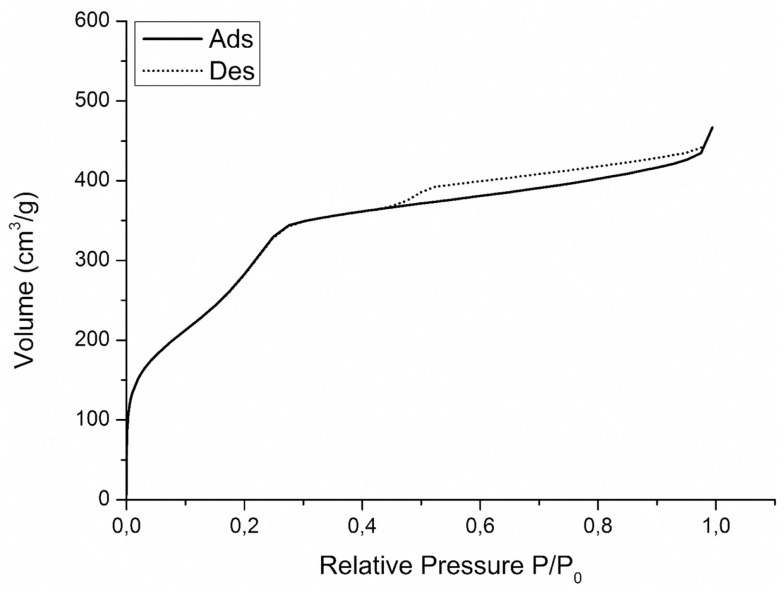
**Nitrogen adsorption desorption isotherms on MCM-41_B**.

**Figure 7:**
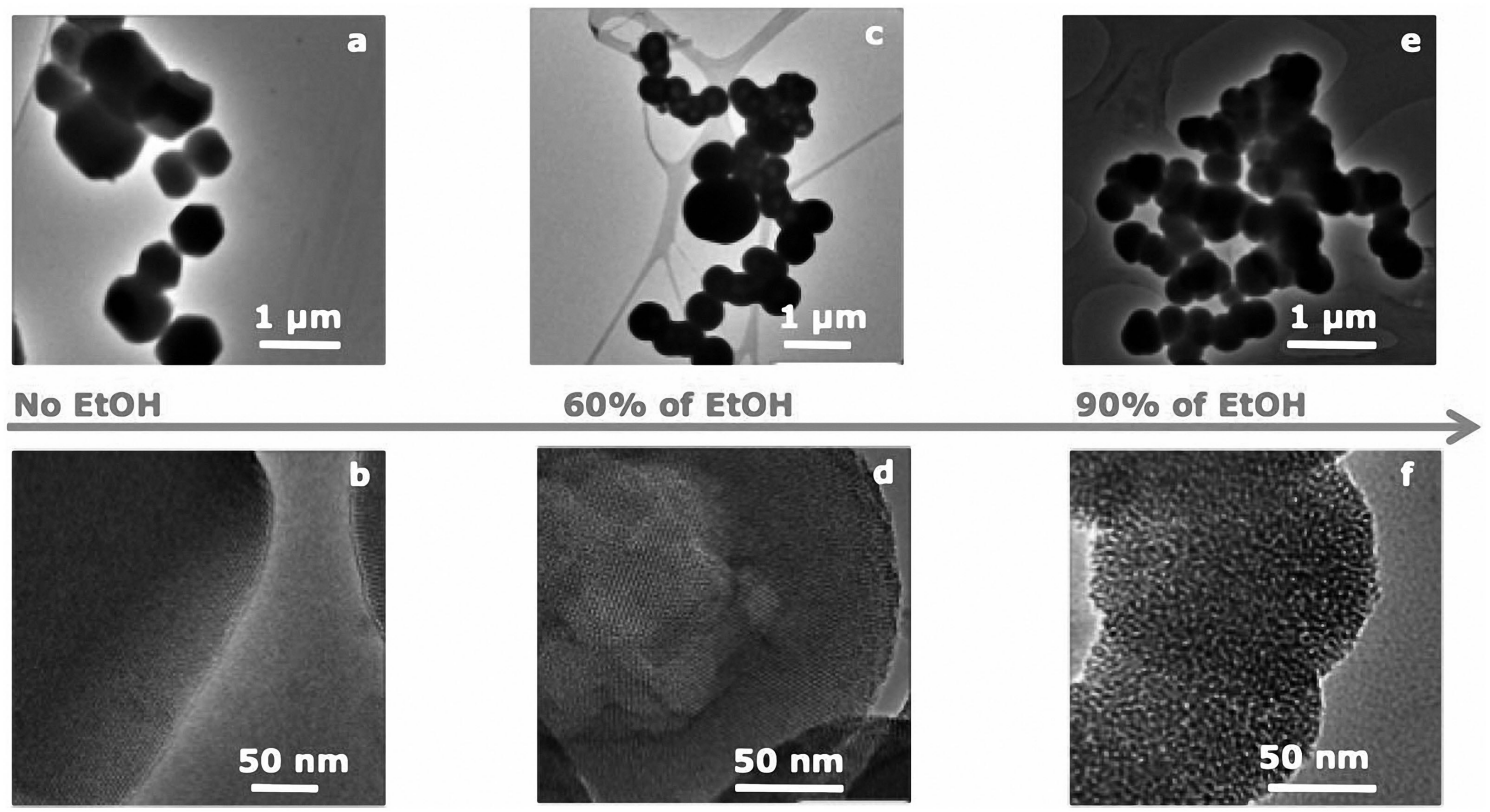
**HRTEM images of the porous structure of samples prepared with different ratios water/ethanol: (a,b) sample MCM-41_A, no ethanol, and 20 min of stirring following the standard synthesis procedure (Zeleňák et al., 2008), (c,d) sample MCM-41_B, 60% ethanol and 20 min stirring, (e,f) sample MCM-41_C, 90% ethanol, and 2 h stirring**.

The nitrogen isotherms of sample MCM-41_B are shown in Figure 6. The isotherms can be classified as type IV isotherms according to the IUPAC nomenclature for MCM-41 (Vallet-Regí et al., 2012a; Zhao et al., 2013), which is typical of mesoporous material with pore diameter in the range of 2–10 nm. MCM-41_B particles were characterized by a specific surface area of 951 m^2^ g^−1^ and a pore volume of 0.24 cm^3^ g^−1^. From Figure 7, it was possible to observe how the different amounts of solvent influenced the final shape and mesostructure of the resulting MCM-41 particles. The particles produced with only deionized water (Figures 7a,b) as solvent were characterized by hexagonal and not spherical geometry (MCM-41_A). Progressively increasing the amount of ethanol as co-solvent, it was possible to produce spherical particles, which exhibited a fairly homogeneous distribution of particle size but reduced mesoporosity order (Figures 7e,f).

### Composite Scaffold System

**Figure 8:**
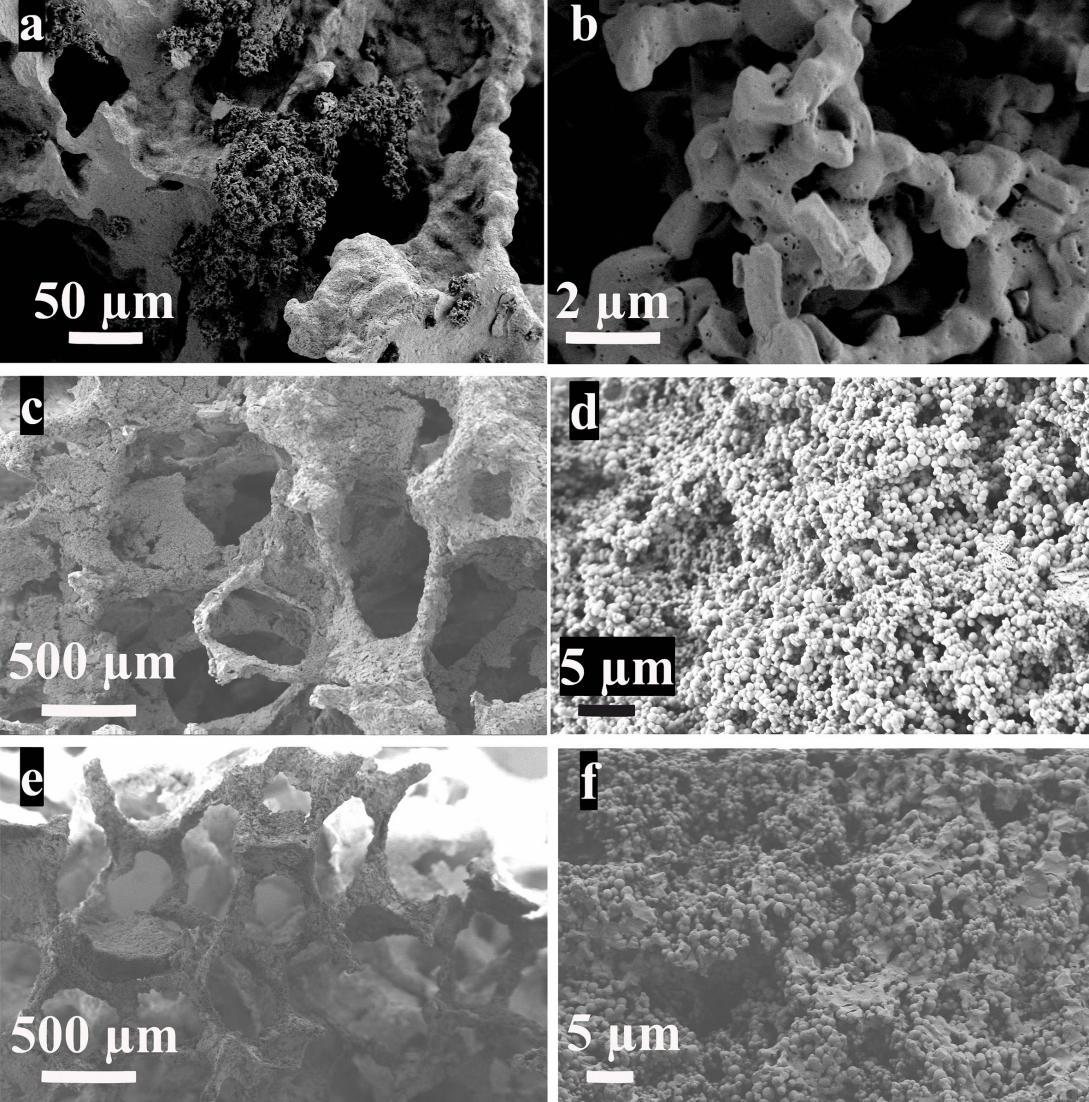
**BG-based scaffolds coated with MCM-41_A (Zeleňák et al., 2008) (a,b), MCM-41_B (c,d), and MCM-41_D (Grün et al., 1999) (e,f)**.

**Figure 9:**
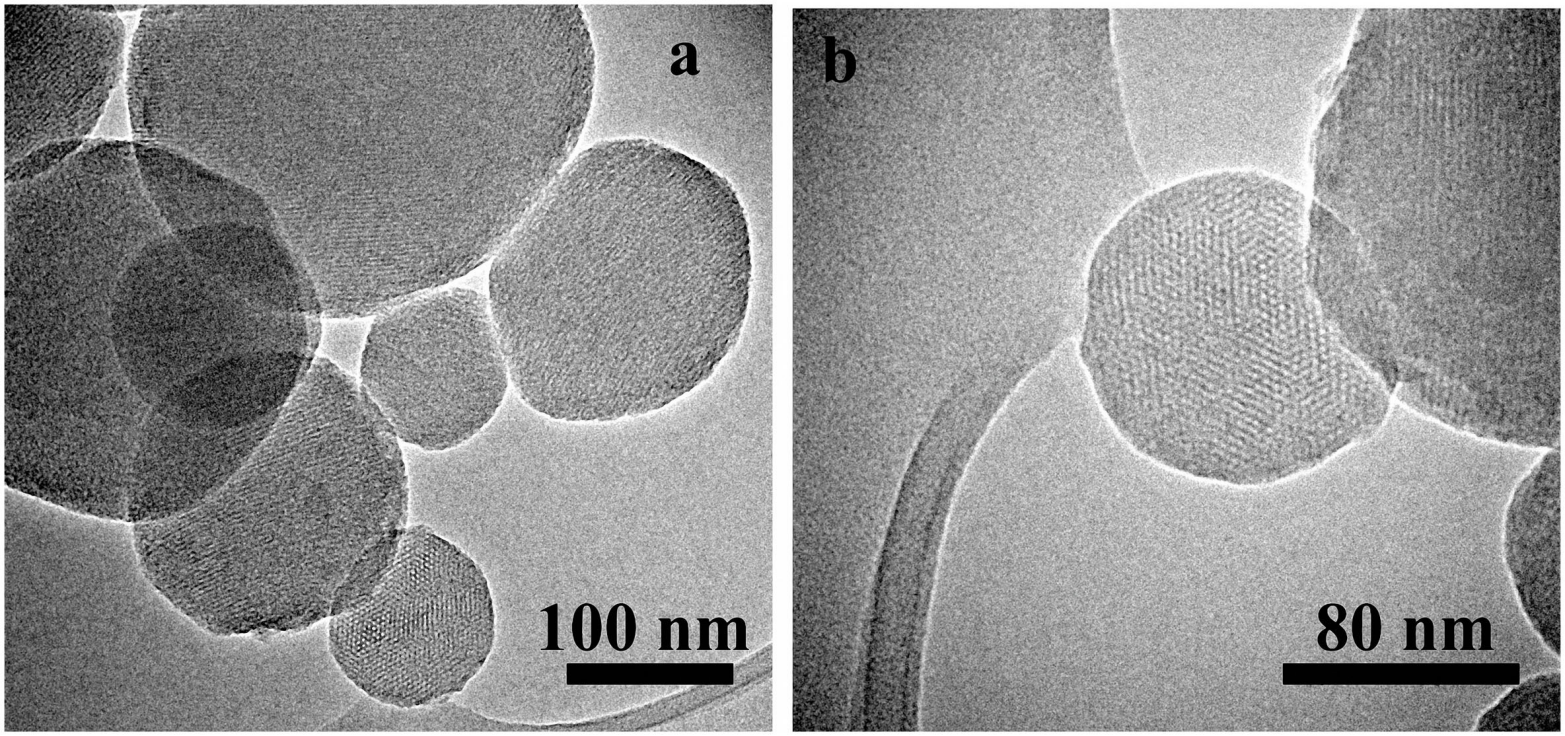
**HRTEM images of the MCM-41_B particles coating of BG-based scaffolds at different magnifications**. TEM images of few MCM-41_B particles **(a)** and HRTEM image of a single particle were the ordered mesoporosity was observed **(b)**.

From SEM analysis, it was possible to observe that the surface of scaffolds was completely coated after immersion in the MCM-41 synthesis batch maintaining an open porosity. In the case of the synthesis solution of MCM-41_A (Zeleňák et al., 2008) (Figures 8a,b), the one without ethanol as co-solvent, the shape of the resulting MCM-41 particles was seen to be completely changed. The presence of the scaffold affected the formation of the particles, probably due to a reduction in the homogeneity of the solution. Moreover, with this solution, it was not possible to obtain a homogenous coverage of the BG scaffold surface. With the synthesis solution of samples MCM-41_B (Figures 8c,d) and MCM-41_D (Grün et al., 1999) (Figures 8e,f), the resulting particles on the surface of the BG scaffolds were still perfectly spherical and they covered completely the surface of the scaffold struts. By means of HRTEM analysis, it was also possible to confirm that the MCM-41_B particles still exhibited ordered mesoporosity, as shown in Figure 9.

### Drug-Release Capability of MCM-41 Particles

**Figure 10:**
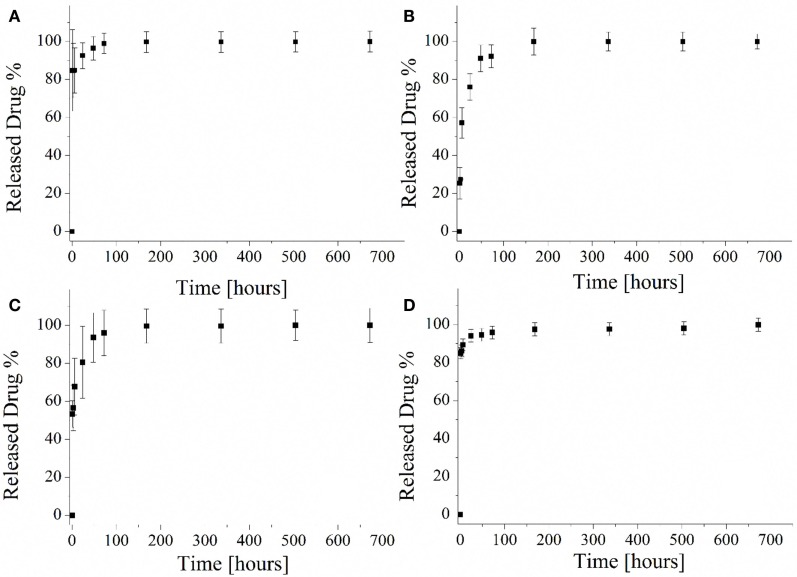
**Ibuprofen released profile from MCM-41_A (A), MCM-41_B (B), MCM-41_C (C), and MCM-41_D (D)**.

The drug-release capability of the different mesoporous silica particles was evaluated and the released profiles are reported in Figure 10. Both samples MCM-41_A (Figure 10A) and MCM-41_D (Figure 10D) were characterized by a burst release and it was confirmed that after 1 h of test, the 80% of the loaded drug was released. The rest 20% of the drug was released within the next 7 days. The particles prepared with solutions MCM-41_B and MCM-41_C showed the best drug-release profile (Figures 10B,C), in terms of lack of uncontrolled burst release. Especially MCM-41_B, which was characterized by high-ordered mesoporosity, did not show any burst release during the first hours of the test. Eighty percent of the loaded drug was in fact released only after 30 h and the rest of the ibuprofen was released within the seven following days. It should be pointed out that ibuprofen solubility in water at 25°C is 21 mg L^−1^ (Vallet-Regí et al., 2001). During the present release test, the highest concentrations of ibuprofen were lower than its solubility, also after the first hours of release. At every time point, 1 mL of fresh PBS was added to every sample to keep constant the PBS volume and for this reason the solution was highly diluted.

### Drug-Release Capability of Composite Scaffold System

**Figure 11:**
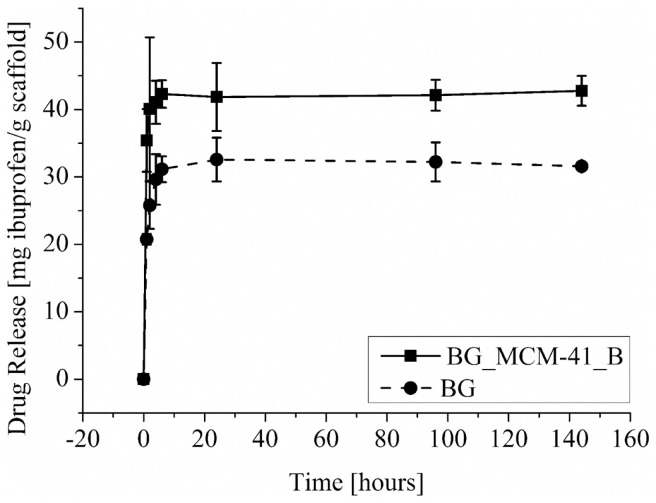
**Drug-release profile from BG scaffolds coated with mesoporous silica particles BG_MCM-41 (■) and uncoated (•)**.

The amounts of released ibuprofen from the BG and BG_MCM-41_B scaffolds are shown in Figure 11. The presence of the mesoporous silica particles increased the drug incorporation capability, but in both cases most of the drug was released during the first hours of the test. The uncoated BG scaffolds were able to uptake 31 mg_IBU_/g_bioglass_, the scaffolds coated with MCM-41_B could uptake 43 mg_IBU_/g_bioglass_.

### Immersion Test

**Figure 12:**
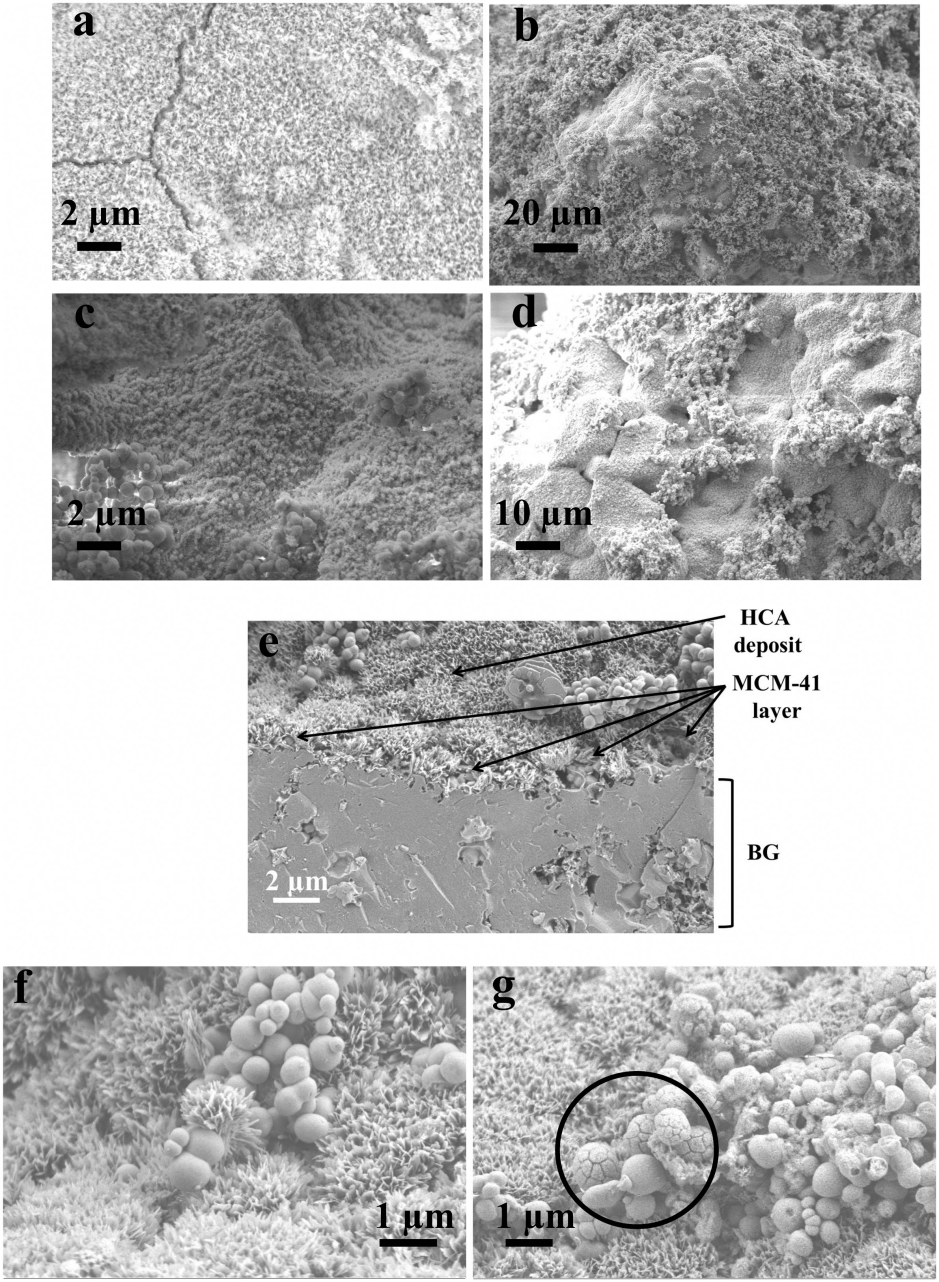
**SEM micrographs of BG-based scaffolds uncoated (a) and coated with MCM-41 (b–d) after 1 week in SBF; BG_MCM-41_B scaffolds after immersion in Tris buffered solution for 10 days (e–g)**.

BG-based scaffolds both uncoated and coated with MCM-41_B particles were immersed in SBF at 37°C. After 1 week of immersion, it was possible to observe that the presence of the silica particles did not affect the bioactivity of the (crystallized) BG struts. In fact on both samples, coated and not coated (Figure 12a), a hydroxycarbonate apatite (HCA) layer formation was seen to form (Figures 12c,d). Moreover, it was possible to confirm the stability of the MCM-41 coating: after 10 days in Tris buffered solution, it was possible to identify the layer of MCM-41 particles covered with the HCA deposit (Figure 12e). The HCA layer was well developed and a deposit was also seen to have formed on the surface of the silica particles (Figures 12f,g, black circle).

## Discussion

One of the most investigated areas in the bone tissue engineering field is related to the development and characterization of mechanically robust and porous 3D scaffolds. The main challenge is the design of a material able to match at the same time the biological and mechanical properties of the natural bone and also release ions or drugs able to reduce the risk of inflammation and infections after the implantation. In a previous work of Mortera et al. (2008), the possibility to increase the functionality of BG-based porous scaffolds was considered using a coating with MCM-41 particles as drug delivery system. In this way, it was possible to combine in a single system the drug uptake and release capability of mesoporous materials with the bioactivity of BG. In the present work, a further development of this idea was presented, improving the homogeneity of the coating, assessing the bioactivity and stability of the composite system BG_MCM-41. Four different solutions were evaluated for the preparation of mesoporous silica particles, and an optimal synthesis procedure was found (MCM-41_B). In fact, by combining two different synthesis pathways, both well known in literature (Grün et al., 1999; Zeleňák et al., 2008), it was possible to obtain particles characterized by spherical shape and high-ordered mesoporosity as confirmed by HRTEM, SAXRD, and Nitrogen adsorption/desorption analysis (specific surface area 951 m^2^ g^−1^, pore volume 0.24 cm^3^ g^−1^). Moreover, the efficiency of the synthesis was increased and the total amount of produced particles obtained per single batch increased up to 60% compared to the previous synthesis procedure: every 500 mL of solution, 4 g of MCM-41_B were produced. MCM-41_B showed the best drug-release profile not exhibiting any burst release during the first hours of the test. The 80% of the loaded drug was in fact released only after 30 h and the rest of the ibuprofen was released within the seven following days. The drug-release times obtained during this work are in agreement with previous studies on drug-release capability of mesoporous silica particles (Vallet-Regí et al., 2001). These novel synthesis solution was used for the coating of 3D BG scaffolds. Due to the high amount of particles produced during the synthesis, a highly homogeneous coating of the scaffolds was obtained. After the coating procedure, the particles were still spherical in shape and also the ordered mesoporosity was not affected. This was an improvement compared to previous works, in which the coatings were not homogeneously distributed on the surface of the scaffolds and the particles were not characterized by ordered mesoporosity (Mortera et al., 2008). The system BG_MCM-41 was assessed to be bioactive. In fact after 1 week of immersion in SBF on the surface of both coated and not coated scaffolds, a layer of HCA was observed. It has been reported in literature that MCM-41 particles are not bioactive and formation of HCA on their surface was not observed after 2 months of immersion in SBF due to the small pore size and the lower concentration of silanol groups (~2 mmol SiOH m^−2^) in comparison to other silica particles such as SBA-15 and MCM-48 (Vallet-Regí et al., 2006), which can act as nucleation sites for the apatite layer. This behavior confirms that MCM-41 particles did not have a negative effect on the bioactivity of the BG scaffolds, on the contrary, combined with BG, they seem to enhance the bioactivity. Moreover, most of the MCM-41 particles were still on the surface of the scaffold and some of them are seen to be also coated with HCA in SEM images (Figures 12f–g). The MCM-41 particles were in fact adhered to the glass surface due to the thermal treatment: the calcination at 550°C is likely to induce softening of the glass (Lefebvre et al., 2007) which should facilitate adhesion of the MCM-41 spheres. For this reason, the MCM-41 particles coating was stable on the surface of the BG scaffold also after immersion in SBF. Moreover, the presence of the particles on the surface of the BG scaffolds increased the drug uptake capability of the scaffolds compared to the not coated ones. Thanks to the ordered mesoporosity and the pore size in the range of 3 nm, MCM-41 particles were thus confirmed to be an optimal drug delivery carrier.

## Conflict of Interest Statement

The authors declare that the research was conducted in the absence of any commercial or financial relationships that could be construed as a potential conflict of interest.
